# Phenotype Diversity of Macrophages in Osteoarthritis: Implications for Development of Macrophage Modulating Therapies

**DOI:** 10.3390/ijms23158381

**Published:** 2022-07-29

**Authors:** Nataliya V. Mushenkova, Nikita G. Nikiforov, Nikolay K. Shakhpazyan, Varvara A. Orekhova, Nikolay K. Sadykhov, Alexander N. Orekhov

**Affiliations:** 1 Unicorn Capital Partners, LLC, 119049 Moscow, Russia; mushenkova@mail.ru; 2 Laboratory of Angiopathology, Institute of General Pathology and Pathophysiology, 125315 Moscow, Russia; nikiforov.mipt@googlemail.com (N.G.N.); drawnman@mail.ru (N.K.S.); 3 Center for Precision Genome Editing and Genetic Technologies for Biomedicine, Institute of Gene Biology, Russian Academy of Sciences, 119334 Moscow, Russia; 4 Federal State Budgetary Scientific Institution “Petrovsky National Research Centre of Surgery”, 117418 Moscow, Russia; nshakhpazyan@gmail.com

**Keywords:** macrophages, inflammation, osteoarthritis

## Abstract

Chronic inflammation is implicated in numerous human pathologies. In particular, low-grade inflammation is currently recognized as an important mechanism of osteoarthritis (OA), at least in some patients. Among the signs of the inflammatory process are elevated macrophage numbers detected in the OA synovium compared to healthy controls. High macrophage counts also correlate with clinical symptoms of the disease. Macrophages are central players in the development of chronic inflammation, pain, cartilage destruction, and bone remodeling. However, macrophages are also involved in tissue repair and remodeling, including cartilage. Therefore, reduction of macrophage content in the joints correlates with deleterious effects in OA models. Macrophage population is heterogeneous and dynamic, with phenotype transitions being induced by a variety of stimuli. In order to effectively use the macrophage inflammatory circuit for treatment of OA, it is important to understand macrophage heterogeneity and interactions with surrounding cells and tissues in the joint. In this review, we discuss functional phenotypes of macrophages and specific targeting approaches relevant for OA treatment development.

## 1. Introduction

Osteoarthritis (OA) affects more than 10% of the global population and represents a growing problem for healthcare systems. It usually manifests above the age of 65 as joint pain and may affect the knees, hips, spine and fingers [1]. Almost half of the adult population in the USA will have symptomatic knee OA by the age of 85, with the highest risk among patients with obesity [2]. Being a progressive condition, OA leads to functional decline and reduced quality of life. To date, there are no effective disease-modifying drugs for this pathology, and current treatment strategies focus on pain relief and viscosupplementation. Most importantly, these treatments are unable to halt disease progression. Joint replacement is a radical procedure performed at advanced stages of the disease [3]. Pain management represents a challenge, as it is often inadequate and associated with safety concerns, especially in elderly patients [4]. Therefore, development of novel treatment approaches to OA is an urgent need and an active topic of current research.

Due to the lack of neutrophils in the synovial fluid and the lack of systemic manifestations of inflammation, OA was not traditionally considered a classical inflammatory arthropathy. However, histopathological studies have identified signs of inflammation within OA synovium, such as cellular hyperproliferation, increased angiogenesis and infiltration of leukocytes suggesting presence of low-grade local inflammation [3]. High levels of plasma proteins, complement components, proteases and cytokines in the synovial fluid and joint tissues are among the prominent signs of joint inflammation. Another sign of inflammation process observed in OA is overproduction of inflammatory mediators by chondrocytes and synovial cells. The two major pro-inflammatory cytokines implicated in OA are IL-1β and TNFα. Presence of synovitis as a characteristic of late-stage disease at the time of total knee or hip replacement was demonstrated by a study that included 104 subjects. Severe inflammation was present in 31%, and only 7 out of the 104 patients had no evidence of synovitis [5]. The degree of inflammation was highly heterogenous between patients, but, nevertheless, was clearly associated with pain and disease progression. Presence of synovitis in OA positively correlates with increased severity of joint symptoms and accelerated destruction of cartilage [6]. Synovitis is associated with a nine- fold greater risk in individuals presenting with painful knee OA [7]. Timing of synovitis and studies in animals deficient for inflammatory mediators suggest that inflammation may be pivotal for joint damage and degeneration [3]. Success with intra-articular injection of glucocorticoids [8] and the usage of non-steroidal anti-inflammatory drugs (NSAIDs) [9] supports the idea of targeting the inflammatory process to treat OA, although numerous attempts to target specific inflammatory mediators have failed [3].

The innate immune system responds to tissue damage via activation of pattern-recognition receptors (PRRs) specific for damage-associated molecular patterns (DAMP). DAMPs are endogenous molecules that are released upon tissue injury. At least four classes of DAMPs were described in OA [10]. They include products of extracellular matrix (ECM) degradation (biglycan, fibronectin, low-molecular-weight hyaluronic acid, tenascin C, etc.), plasma proteins that exude at the sites of inflammation-induced vascular leakage (such as a1 microglobulin, α2 microglobulin, fibrinogen, vitamin D-binding protein, Serum amyloid A), intracellular alarmins (for example, HMGB1 and the S100 family of proteins), and microscopic crystals released from injured cartilage (basic calcium phosphate, calcium pyrophosphate dihydrate, and uric acid) [3]. DAMPs are sensed by a class of pattern recognition receptors (PRRs) known as Toll-like receptors (TLRs). Among the ten types of TLRs that are functional in humans, TLR1-TLR7 and TLR9 are most likely to be implicated in OA pathology, since their overexpression was observed in inflamed joints [11]. In synovial fluid from OA-affected joints, TLR4 was also present in the soluble form (sTLR4) and was associated with disease severity, making sTLR4 a possible biomarker [12]. The role of specific TLRs and their use as therapeutic targets are currently unclear due to the mixed results from in vivo studies [3,13]. Moreover, important differences between TLR systems in mice and humans have been described [14]. There are also joint-specific patterns of TLR expression, particularly between hand and knee, suggesting differential immune mechanisms operating in large and small joints [15]. DAMPs recognition by TLRs results in the activation of NF-kB- and AP-1-driven transcriptional programs, production of cytokines, chemokines, growth factors, and matrix proteases that coordinate cellular proliferation and tissue remodeling responses. In chondrocytes, TLRs activation may lead to a direct apoptotic response [15]. Sustained protease-mediated degradation of cartilage and meniscus tissue and mechanically induced tissue damage generate a sustained source of DAMPs leading to inflammation and tissue destruction. The main types of innate cells responsible for DAMP recognition in joints are macrophages and mast cells. The role of mast cells in the OA inflammatory cycle remains poorly understood and is a subject of ongoing studies [16,17,18,19].

Macrophages are well-known cellular mediators of innate immunity and tissue remodeling that actively participate in the healing response following injury [20]. Furthermore, macrophages are also capable of bridging and instructing the adaptive immune system via various secretory mediators and direct antigen presentation [21]. Depending on the local cytokine milieu, macrophages undergo polarization to different phenotypes with opposing roles in inflammation and tissue healing. Pro-inflammatory M1 are induced, among other factors, by IFN-γ, TNF-α, GM-CSF and LPS, and produce proinflammatory cytokines IL-12, IL-6, IL-1β and TNF-α [22]. Known markers of M1 cells are CD86, CD40, inducible nitric oxide synthase (iNOS)/NOS2, CCR7, CD11c, and CD16/32 [23]. In the synovial tissue, M1 macrophages promote destructive processes associated with inflammation. It was shown that T cell and monocyte infiltration, as well as synovial and systemic concentrations of inflammation mediators, appear to be less pronounced in OA as compared to rheumatoid arthritis (RA) [3]. However, low-grade inflammation is clearly present and plays a role in the disease pathogenesis, highlighting the possibility that OA- and RA-associated inflammation may be regulated by distinct mechanisms and require different treatment approaches.

M2 macrophages differentiate in response to Th2 cytokines, such as IL-4 or IL-13 and produce anti-inflammatory cytokines IL-10, TGF-β, IL-1 receptor antagonist (IL-1Ra). These cells promote angiogenesis and tissue repair [22]. Furthermore, several subtypes of M2 macrophages have been identified depending on the differentiation signal Typical markers of M2 macrophages are CD163, CD206, Arg1, and CCL22 [23].

The concept of M1/M2 polarization was revised due to advances in next-generation sequencing and transcriptomic analysis that unraveled more complex heterogeneity of macrophage population. 

According to current understanding, synovial M1 macrophages differentiated in response to DAMPs that are present in osteoarthritic joints contribute to cartilage damage and bone alteration. This was supported by in vitro studies showing that depletion of CD14+ macrophages from synovial cell cultures results in a reduction of proinflammatory cytokines, as well as MMPs and aggrecanase enzymes able to degrade joint cartilage [24]. Furthermore, depletion of CCL2 chemokine or its receptor that are involved in macrophage infiltration was associated with reduced pain severity and structural disease in a model of joint de-stabilization [25]. However, total depletion of joint macrophages was shown to enhance OA pathology. The importance of targeting specific population of macrophages is currently evident. Classical M1/M2 concept fails to describe the real phenotypic heterogeneity of joint macrophages, as well as multiple roles of these cells not only in cartilage destruction, but also in pain and cartilage regeneration process [26].

## 2. Macrophage Infiltration Pattern in OA

The synovial membrane is responsible for production of synovial fluid that lubricates and nourishes chondrocytes and represents the main reservoir of macrophages within the joint. Normal synovium consists of two distinct layers that also serve as distinct macrophage niches within the synovial tissue. One is the intimal lining layer important for barrier function with two to three layers of macrophages and fibroblast-like synoviocytes (FLS). The other layer is the synovial sublining layer, consisting of fibrous connective tissue and blood vessels. The presence of macrophages in the sublining layer is minimal, but substantially increases during inflammation due to the massive infiltration of monocytes from circulation. Therefore, synovial macrophages can be tissue-resident or non-resident, and differ in localization, potential for self-renewal, surface markers [21], functional phenotype [23] and the role in OA pathogenesis.

Presence of activated macrophages in patients with knee OA was studied directly in vivo using ^99m^Tc-EC20 (etarfolatide) imaging agent, that selectively binds to folate receptor β (FRβ) on activated, but not resting macrophages or other immune cells. Expression of FRβ was described previously for both M1 and M2 macrophage phenotypes. The majority of joints (76%) were positive for activated macrophages in at least one knee region; the main structures for etarfolatide uptake were joint capsule (66%), synovium (52%), and subchondral bone (16%) [27]. The level of activated macrophages in knee joints positively correlated with radiographic OA severity and clinical symptoms, suggesting they may be an important pathogenic factor [27,28]. CD14 and CD163 were suggested as possible biomarkers for activated FRβ+ macrophages in OA joints. Both these macrophage markers exist in soluble form and can be measured in serum and SF, though only serum sCD163 was significantly associated with activated macrophages in the knee joint capsule and synovium [28]. CD163 is a known M2 marker, though it was also reported to be present in M1 macrophages [29]. CD14 expression was demonstrated to be higher in M2 macrophages than in M1 subtype. The association of soluble CD163 and CD14 with the abundance of activated macrophages suggests a role of M2 macrophages in OA pathogenesis.

The immune cell landscape was studied in detail for knee OA using Cell-type Identification by Estimating Relative Subsets Of known RNA Transcripts (CIBERSORT) technique. This method can distinguish 22 immune cell types at once and apply signatures from ~500 marker genes to quantify the relative fraction of each cell type, thus possessing a high-resolution power [30]. M2 macrophages were predominant in the synovial tissue of OA patients, constituting 30.1% of cells, while resting T cell CD4 memory cells accounted for 23.9%, and activated NK cells–for 16.2%. Though these data are close to the immune profile of the normal synovium (26.8%, 24.1%, 15.0%, respectively), the observed increase in proportion of M2 macrophages was statistically significant. Computationally estimated cell composition in synovial tissue based on publicly available transcriptomic data also confirmed increased M2 macrophage content in OA synovium (26.9% in OA vs. 21.9% in healthy joints), with a significantly higher percentage of monocytes than in healthy controls (5.9 vs. 3.4%) [21]. M1 macrophages constitute the minor population, close to 1% of infiltrated cells in both cases. Adipocytes were shown to be the cell population that undergoes the most significant change, with a three-fold reduction in inflamed synovium [31]. Further studies should clarify the significance and possible mechanisms of such changes.

**Figure 1:**
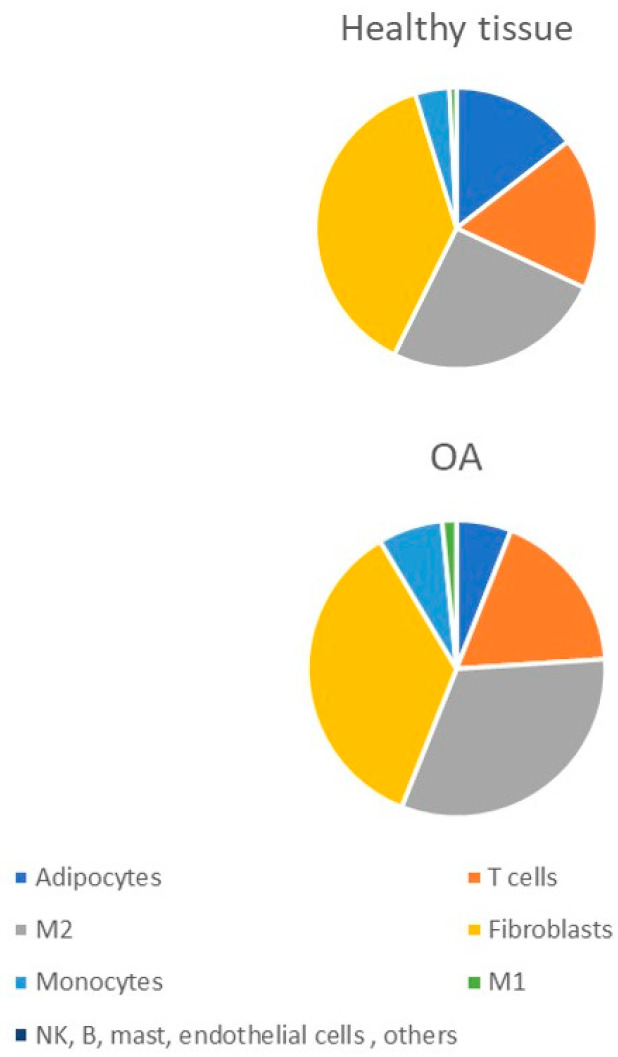
Estimated cell compositions in human synovial tissue based on publicly available transcriptomic data (according to data from [31]).

Comparison between cellular composition of synovium in OA and RA patients have shown completely different patterns (Figure 1). The percentage of monocytes was much higher in RA (12.7% in RA vs. 5.9% in OA). RA synovial tissue also had a higher percentage of M1 macrophages (4.1% in RA vs. 1.2% OA) and a lower percentage of M2 macrophages. This fact indicates that RA and OA are probably characterized by different inflammatory processes and explains the known responsiveness of RA to anti-TNFα treatment as both cell populations upregulated in RA synovium, monocytes and M1 macrophages, are known to be capable of expressing high levels of TNFα. Data gained with transcriptomic studies provide different results than immunohistochemistry evaluation of synovial macrophages, showing that M1 (defined as iNOS1-positive cells) is the predominate macrophage population in OA synovium and undergoes a three-fold increase in comparison with normal tissue [32].

Macrophages represent a minority among the immune cells in the OA subchondral bones, with the most abundant infiltrating immune cell types being CD8, CD4 T and mast cells. There is almost no infiltration of immune cells in the meniscus and cartilage of the knee [30]. There may be transient macrophage infiltration of ligament and tendon tissues induced by damage. In this case, balanced M1/M2 response is crucial for proper healing process. Pro-inflammatory macrophages play an important role in the acute inflammatory phase post-injury by removing tissue debris and matrix remodeling. However, excessive matrix degradation is detrimental, resulting in decreased tensile strength of the healed tendon [33]. Furthermore, ligament or tendon injury may result in macrophage infiltration of other surrounding joint tissues, leading to a local pro-inflammatory response [21].

The infrapatellar fat pad (IFP), or Hoffa’s pad is an adipose tissue depot present in the knee joint. It is densely vascularized and innervated and may be an important reservoir of macrophages [21]. IFP in OA appears to be more inflamed and vascularized compared to non-OA controls [34] with an immune cell profile comparable to that of the synovium [21]. Macrophages present in IFP have a mixed pro- and anti-inflammatory phenotype. The existing evidence indicates that CD206^+^ cells (a marker of M2) are involved in the inhibition of catabolic processes. At the same time, their pro-fibrotic and pro-angiogenic activity may have deleterious effects. IFP undergoes hypertrophy in OA patients and becomes an important source of catabolic, pro-fibrotic factors, adipokines and M1-polarizing cytokines, such as IL-6 [21]. IL-6 is considered as one of the most represented cytokines involved in OA inflammation [35]. The expression of IL-6 and its receptor sIL-6 in the IFP is high, indicating that IL-6 may affect other joint components in a paracrine manner. IL-6-dependent signaling is involved in cartilage degradation and pain [35]. IFP has gained increasing attention as a potential therapeutic target for pain management in OA [34]. The crosstalk between inflamed IFP and surrounding tissues and its role in OA pathogenesis is an interesting area of research [36].

Furthermore, macrophages and monocytes are often detected in the synovial fluid and were shown to positively correlate with joint stiffness, pain and reduced quality of life [37].

## 3. Functional Diversity of Synovial Macrophages in OA

Despite the proposed pathogenic role of activated macrophages in OA, their systemic depletion was shown to be detrimental. In destabilization of medial meniscus (DMM)-induced OA model in obese MaFIA (Macrophage Fas-Induced Apoptosis) mice, total macrophage decrease led to severe synovitis and failure to relieve cartilage deterioration [38]. Depletion of joint macrophages in the same model also did not alleviate OA progression [38], but instead increased the infiltration of CD3+ T cells and neutrophils into the affected joint, with potentiation of synovitis and systemic inflammation.

In OA synovium and cartilage, cytokines associated with M1 macrophages are known to stimulate pro-catabolic mediators, such as aggrecanases and MMPs [20]. Imbalance of M1 and M2 populations was described by Liu et al. [39] in human knee OA and was shown to positively correlate with Kellgren–Lawrence grade. This study showed a more than two-fold increase in synovial cells with CD11c marker (defined as M1 macrophages) in OA samples with a concomitant decrease in CD206 or M2 cells [39]. However, this observation of M1/M2 imbalance should be considered with caution, since CD11c is also a marker of monocytes and its increase may reflect growing monocyte infiltration of the synovium. CD11c is also expressed by granulocytes, dendritic cells and B cell subpopulations. The level of macrophage pro-inflammatory polarization might vary in different OA subtypes. For example, OA patients may be divided into two subgroups depending on the level of Prostaglandin E2 (PGE2) production in IFP [40]. The IFP from high PGE2 OA group showed greatly enhanced expression of M1 cytokines IL-6 and IL1β in comparison to PGE2-low group. Higher inflammatory profile was associated with a 3.7-fold lower gene expression of CD163 (an M2 macrophage marker).

In end-stage OA, both M1- and M2-like macrophages are present in the joint synovium and IFP, the functional significance of which remains to be evaluated [20]. Mice with either M1- or M2-enhanced macrophage polarization were used to evaluate the positive and negative effects of polarization bias in two models of knee OA, collagenase and meniscal destabilization with high and low levels of synovitis, respectively [32]. Pro-inflammatory M1 bias was associated with greater synovial inflammation and cartilage pathology, as compared to mice with predominant M2 polarization. The protective effects of M2 bias were evident in both models, though at late time points [32]. These data are in accordance with the results obtained in OA models deficient for IL-4, a central M2-driving cytokine, or IL-4 signaling. These mice developed exacerbated cartilage damage and osteophyte formation relative to wild-type controls. IL-4 promoted an immunomodulatory microenvironment with M2 shift of joint macrophages that results in efficient clearance of cartilage debris and inhibition of osteoclast differentiation [41].

DMM-induced OA is a well-established OA murine model that has been widely used to study the macrophage alterations in OA [42]. The number of M1 macrophages (defined as F4/80^+^ CD86^+^ CD63^−^ or F4/80^+^ CD11c^+^ iNOS^+^, depending on the study) was significantly increased in the synovium six weeks after DMM [43,44], accompanied by sustained downregulation of CD206^+^ F4/80^−^ CD11c^+^, or M2 macrophages [44]. The qPCR studies revealed up to 2000-fold upregulation of iNOS with a 30–35-fold fall in M2 transcripts thus showing a clear M2 to M1 shift. Another study in DMM model showed a less pronounced M1 to M2 shift. Although upregulation of CD64^+^ iNOS^+^ staining was observed in damaged knees, CD206 staining, reflecting M2-like activation, was also statistically higher in damaged vs. intact knees. Similar trends in the change of macrophage populations were also found in a more severe OA model, anterior cruciate ligament transection (ACLT) [45]. In collagenase-induced osteoarthritis (CIOA) in mice that is characterized by a strong inflammatory pattern, a four-fold sustained increase in the number of F4/80^+^ iNOS^+^ cells was shown in the synovium at 7 and 28 days after induction, accompanied by insignificant changes in the amount of M2 cells (F4/80^+^ CD206) [32]. In dogs with cruciate ligament rupture, M1 shift occurs early, and OA lesions are characterized by increased M1 (CCR7^+^ iNOS^+^ CD68^+^)/M2 (CD163^+^ Arg1^+^ CD68^+^) ratio [46].

The use of animal models of pro-inflammatory macrophage transition in induced OA is limited. Data gained in naturally occurring equine OA may be more relevant for human situation. Comparison of cytokines and macrophage markers in the synovium of healthy equine carpal and metacarpophalangeal (MCP) joints and those with moderate OA had not provided a conclusive picture [47]. Cytokine profiles differed between carpal and metacarpophalangeal joints. Pro-inflammatory shift with increase in IL-6 level and statistically significant decrease in IL-10 level (without significant upregulation of TNFα and IL-1) observed in MCP was not confirmed for carpal joints [47]. This difference may be connected with a higher level of mechanic stress and damage in the case of MCP. Markers used to define M1- (CD86) and M2-like (CD206 and IL-10) macrophages were similarly expressed in both healthy and OA-affected groups. 

Classical M1/M2 paradigm might not be appropriate when studying early- and late-stage events in OA [23]. Known macrophage populations that have been identified in human knee OA are presented in Table 1. A flow cytometry study compared the population of immune cells in the synovial tissue from OA patients undergoing total knee replacement and inflammatory arthritis patients [48]. The study confirmed the predominance of macrophages in the OA-inflamed synovium, in contrast to inflammatory arthritis patients with a T cell-dominant pattern. The macrophage content was highly variable between patients, with HLA-DR^+^ CD14^+^ calls comprising from 1 to 50% of all CD45^+^ cells [48]. Macrophages in OA exhibited an activated phenotype with the expression of folate receptor-2 and CD86 and high phagocytic capacity. Transcriptomic analysis of synovial macrophages identified two distinct OA subgroups: one with a proliferative and pro-inflammatory phenotype, closely resembling the inflammatory arthritis group (named iOA), and the other-characterized by cartilage remodeling genes (named classical or cOA) [48]. Gene expression analysis revealed that 155 genes were differentially expressed between the cOA and iOA subgroups. In contrast to cOA, the iOA group displayed significantly higher level of Ki67 marker of proliferation, as well as other proliferation-associated genes (*E2F8*, *CDT1*). Classical cOA subtype was defined by high level of IGFBP5, which is known to be associated with negative regulation of inflammatory mediators, as well as genes of cartilage degradation and repair (*HTRA1*, *EFEMP1*). The study attempted to match gene expression profiles of synovial macrophages with distinct human knee OA endotypes, demonstrating that proliferating macrophage signature can help identify OA patients in whom disease progression is associated with synovial inflammation. Surprisingly, neither subgroup clearly corresponded to M1 or M2 phenotypes, suggesting the presence of mixed phenotypes of macrophages. Analysis of gene expression of M1-like and M2-like markers in human end-stage OA synovial tissue and healthy control synovium using microarray analysis revealed similar upregulation of both M1 (CD86, CCL3, and CCL5) and M2 (CD206, IL-10, and IL-1Ra) markers. This finding did not support the hypothesis of phenotype shift [49]. Single-cell transcriptome analysis of resident macrophages isolated from meniscal tissue of healthy donors and patients with OA showed downregulation of both M1 markers, such as SIGLEC1, and M2 markers, such as CSF2, indicating that macrophages in the OA state polarize towards directions other than the M1/M2 division [50].

A study published by Kraus et al. described cellular and transcriptional heterogeneity of synovial tissues from OA patients and identified 12 synovial cell populations [27]. Among them were two distinct macrophage populations: pro-inflammatory (I-MΦ) and immune-regulatory (IR-MΦ). Both populations had similar levels of HLA-DRA, CD14, and CD163. I-MΦ were characterized by higher levels of IL1A, IL1B, IL-6, TNF, CCL2 and CCL3, while upregulation of immunoregulatory markers STAB1, TNXIP, and CD169 was shown for IR-MΦ cells. The study demonstrated that I-MΦ macrophages are not the only possible source of pro-inflammatory cytokines in the synovium, since dendritic cells and fibroblasts also expressed high levels of IL1-β and TNF-α. Cell surface proteins HLA-DQA1, HLA-DQA2, and TLR2 were more abundantly expressed in I-MΦ than in IR-MΦ cells [30].

Culemann et al. described a subpopulation of synovium-resident CX3CR1+ macrophages present in murine knee joints. This subset expressed tight junctional protein markers usually associated with that of endothelial cells (F11r, ZO-1 and Claudin-5), as well as genes involved in planar cell polarity, including *Fat4* and *Vangl2*. The subset was maintained by proliferation of the Ki67 ^+^ CX3CR1^−^ interstitial macrophage population [52]. CX3CR1+ macrophages covered the lining of collagen VI-expressing synovial fibroblasts forming a tightly connected structural barrier between the synovial capillary network and the intra-articular space [52]. In murine joints they constituted 40% of the total synovial macrophages under steady-state conditions. The induction of inflammation in K/BxN serum-transfer arthritis (STA) model resulted in distortion of the barrier. Furthermore, the onset of STA was accompanied by the appearance of additional clusters of mononuclear phagocytes that displayed the signature of monocyte-derived macrophages (mostly CCR2^−^ and Ly6c2^−^ expressing cells). These clusters expanded during the progression of arthritis and displayed a pro-inflammatory activation profile, including the expression of IL1-β, while CX3CR1^+^ still maintained their immune-regulatory phenotype. Both systemic and local depletion of CX3CR1^+^ lining macrophages, as well as disruption of tight junctions upon the injection of Claudin 5 peptidomimetics resulted in increased influx of polymorphonuclear leukocytes and exacerbation of experimental arthritis, proving protective function of CX3CR1^+^ population. Transcriptional profiling of CX3CR1^+^ macrophages revealed the expression of immune-related genes (*TREM2* and *VSIG4*), as well as high levels of receptors that mediate the clearance of apoptotic cells, such as AXL and MFGE8. Interestingly, the transcriptional profile of CX3CR1^+^ cells matches that of a subset described in human OA by Zhang and colleagues who studied sc-RNA sequencing data sets [53]. They identified four transcriptionally distinct subsets, including SC-M1 or IL-1B^+^ pro-inflammatory monocytes (with *IL-1B*, *NR4A2*, *HBEGF*, *PLAUR* and *IFITM3* expressing pattern), SC-M2 or NUPR1^+^ monocytes (*VSIG4*, *GPNMB*, *MERTK*, *NUPR1*, *CTSK*, *HTRA1* genes), SC-M3 or C1QA^+^ monocytes (*C1QA*, *MARCO* genes), and SC-M4 or IFN-activated monocytes, expressing genes induced by type I and II IFN (*LY6E*, *IFITM3*, *IFI6*, *SPP1*). The SC-M2 subset, which expresses *VSIG4* and is upregulated in OA, may be similar to mouse CX3CR1^+^ resident macrophages [52]. The results of confocal immunofluorescence microscopy and flow cytometry of human synovial tissue samples confirmed the presence of dense macrophage lining, consisting of macrophages expressing tight-junction proteins and TREM2 that resemble the murine CX3CR1^+^ population. TREM2^+^ MHCII^−^ population was also identified in synovial samples from patients with OA [52]. 

MerTK expression was shown to be characteristic of one of the macrophage clusters in OA that was marked by a subset of genes involved in phagocytosis, such as *ITGB5* and *ADORA3* [51]. Genes expressed by this cluster were enriched in OA tissues as compared to RA. Comparison of single-cell gene sets between MerTK^+^ cluster and human blood–derived macrophages activated by diverse stimuli revealed it was not driven by classical M1 or M2 stimuli, indicating a distinct macrophage phenotype that differentiates in the synovial tissue environment [51].

Macrophage heterogeneity beyond the classical M1/M2 classification is also a typical feature of RA [51,54]. Certain macrophage populations were associated with clinically distinct states of RA. Single-cell RNA sequencing revealed nine synovial macrophage clusters that could be classified into four subpopulations: TREM2^+^, FOLR2^high^, HLA^+^ and CD48^+^. MerTK^+^ TREM2^+^ and MerTK^+^ FOLR2^+^ macrophages were predominant populations in healthy tissue that expressed genes associated with inhibition of T effector cells. Sustained remission was associated with the increase in MerTK^+^ FOLR2^high^ LYVE1^+^ macrophages with a proposed role in tissue homeostasis. By contrast, active RA was associated with increased proportions of MerTK^−^ CD48^−^ SPP1^+^ and MerTK^−^ CD48^−^ S100A12 subpopulations characterized by a pro-inflammatory transcriptome pattern [54]. RA-predominant macrophage subpopulation was defined by the expression of *HBEGF* and classic M1 (*IL1B*) as well as M2 -related genes (*MERTK*, *PLAUR*). According to in vitro data, transcriptional signature of HBEGF^+^ population was shaped in the presence of both inflammatory cytokines and synovial fibroblasts, suggesting fibroblasts are an important component of synovial niche.

Overall, it is currently well established that increased macrophage recruitment correlates with disease activity and clinical signs of OA. However, comprehensive classification of synovial macrophages related to disease stages and activity of OA is currently lacking, reflecting a significant gap in our understanding of the role of macrophages in OA pathogenesis. Studies of cytokine patterns and macrophage phenotypes in OA joints failed to demonstrate clear M1 pro-inflammatory polarization. Furthermore, lack of standardized and specific markers for identifying different subtypes of macrophages makes comparison of results of different studies challenging [23]. In human OA and natural animal models, a mixed set of M1 and M2 markers can be seen. This can be explained by the fact that OA is a heterogeneous disease, with various contributions of inflammatory and immunopathological mechanisms predominating in different endotypes. To clarify the role of macrophage functional polarization, it is important to develop classification of disease subtypes and use adequate stratification of patients. It is also important to keep in mind that association should not be interpreted as causation, as upregulation and activation of specific macrophage populations may just reflect the failure of homeostatic mechanisms and not the pathogenic role. It is important to understand synovial microenvironment in OA and specific macrophage polarizing signals present in inflamed joints.

## 4. Macrophage Polarization Signals and Modulation of Functional Phenotypes in OA

Despite the absence of clear M1/M2 shift during OA development, several OA-specific polarizing signals present in affected joints have been described. It was shown that the synovial fluid of patients with OA possessed a proinflammatory environment [55]. Incubation of knee synovial fluid of patients with knee osteoarthritis (KOA SF) with PBMC-derived macrophages clearly had an M1-promoting effect [55]. At the same time, equine bone marrow mononuclear cell (BMNC) cultures in autologous normal (SF) and inflamed synovial fluid (ISF) were neither inclined to the M1 nor M2 phenotype, but exhibited a mixed phenotype with increasing counts of IL-10^+^ macrophages and decreased IL-1β production [56]. 

Alarmins S100A8 and S100A9 were shown to be implicated in murine and human OA, as well as in multiple rheumatic diseases. They can be detected both in serum and SF of patients with OA. Up to 10–11-fold increase of S100A8 and S100A9 mRNA was observed in OA synovium compared with control tissue from patients with acute joint trauma [57]. M1-like macrophages were confirmed to be the main producers of S100A8 in the synovium [49]. S100A8 and S100A9 promoted synovitis, cartilage degradation, and osteophyte formation via activation of TLR4 and canonical Wnt signaling. Alarmins induced a pro-inflammatory cytokine response with upregulation of IL-6, IL-8, and TNF-α confirmed at the protein level both in M1 and M2-like macrophages (though less pronounced in M2 cells), with induction of matrix-degrading MMPs only in the M1 population. Thus, alarmin production by synovial M1-like cells may resemble a self-reinforcing mechanism of pro-inflammatory polarization in synovial tissue. Extracellular high mobility group box 1 (HMGB1) is a known alarmin released by stressed and dying cells in a variety of inflammatory conditions. It is secreted by FLSs in response to inflammatory factors. HMGB1 level in synovial tissues increases during KOA progression and correlates positively with synovitis. HMGB1 sensing by RAGE and TLR4 receptors leading to strong M1 activation was described in several pathologies. Its relevance for macrophage polarization in OA warrants further study [58].

Basic calcium phosphate (BCP) crystals, comprised mainly of hydroxyapatite, are uniquely associated with OA and have been identified in 100% of cartilage and 50% of synovial fluid samples in patients with late-stage OA. Their concentration correlated with the severity of histological and radiographic OA [59]. BCP crystals polarize primary human macrophages towards an M1-like phenotype with upregulation of CXCL9, CXCL10, IL-8, CD86, and CD40, and downregulation of M2 markers MRC1 and CCL13. In accordance with M1 shift, concomitant decreases in phagocytic capacity and glycolytic metabolic switch were observed. Though the primary way of macrophage pro-inflammatory activation is through PRRs signaling, additional NLRP3 -inflammasome pathway may also be active in the context of OA [60]. Uric acid recognition was described as one of the inflammasome activating signals relevant for OA. Uric acid concentrations in SF correlated with the concentrations of IL-18 and IL-1β, known to be produced by activated inflammasomes, and SF IL-18 was positively associated with OA severity measured by both radiograph and bone scintigraphy [60].

Human synovial membranes produce a broad spectrum of antimicrobial peptides. It was shown that, under inflammatory conditions, their expression pattern alters [61]. Although little is known about the role of distinct antimicrobial peptides in OA development, it was shown that alpha-defensin-1 can act as an M1 to M2 polarization signal in vitro. In co-culture, alpha-defensin-1-polarized macrophages modified OA chondrocytes to regenerative-like state based on the expression of *COL2A1*, *ACN*, *MMP3*, *MMP13* and *ADAMTS5*. Possible protective role of alpha-defensin-1 in OA was confirmed in vivo in a meniscal/ligamentous injury (MLI)-induced rat model. Intra-articular alpha-defensin-1 injections decreased severity of cartilage damage and synovitis, suggesting possible therapeutic utility of this approach [62].

LPS, an outer-membrane component of Gram-negative bacteria, is regarded as a classical M1 polarizing signal, which primes the proinflammatory response via activation of TLR4. The Gram-negative flora of the terminal ileum and large intestine constitute a large reservoir of LPS, small amounts of which may be absorbed into the circulation resulting in low-grade inflammation. Gut-derived LPS have been considered as potential factors in the development of many chronic diseases, including RA, inflammatory bowel disease, type 1 diabetes mellitus, atopy and obesity [63]. LPS and the microbiome are currently unexplored factors in the pathogenesis of OA. However, available data on the positive association of SF LPS with the abundance of activated macrophages in the synovium, as well as symptoms and radiographic abnormalities of knee OA (osteophyte severity, joint space narrowing severity and total WOMAC score) support a role for LPS in the pathogenesis of OA [64].

Components of cartilage matrix and products of its degradation are also important modulators of macrophage phenotype and may act as enhancers of LPS-induced response. Lumican (LUM), a major extracellular matrix glycoprotein in articular cartilage, is one of the macrophage-polarizing signals in OA. Cartilage degradation releases LUM, leading to accumulation of LUM and its fragments in SF at supraphysiological levels [65]. It was found to potentiate LPS inflammatory responses in a TLR4-dependent manner. In addition to the numerous effects on chondrocytes, LUM co-stimulation was shown to shift macrophage polarization towards M1 phenotype, with downregulation of M2 markers. By contrast, another matrix component of collagen II was shown to induce M2 polarization by increasing the expression of M2-related genes (*MR*, *Arg1*, *Fizz1*, and *Ym1*), as well as pro-chondrogenic cytokines (TGF-β and IGF).

Cell-to-cell communication of macrophages with various types of cells present in joints is an important constituent of the joint niche. Chondrocytes, fibroblasts, nerve and adipose cells are among the cells that are known to shape macrophage functionality. Chondrocytes may participate in directing macrophage polarization not only by producing proteases that degrade the cartilage matrix, but also via the release of exosome-like vesicles (EVs). Exosomes are secreted vesicles that can mediate communications between different cells and participate in immune homeostasis and stress response [66]. Exosomes from chondrocytes treated with IL-1β were shown to increase the production of mature IL-1β by macrophages, reflecting M1-like activation. Intra-articular injection of EVs aggravated cartilage erosion and synovitis in DMM-induced OA in mice [67]. Exosomal crosstalk between macrophages and chondrocytes is bidirectional. M1 macrophages produce EV enriched with miR-1246, a known activator of the Wnt/β-catenin pathway and chondrocyte inflammatory response [68]. Presence of miR-1246-rich EVs in synovial fluid of patients with OA has been confirmed [67]. 

Interactions with synovial fibroblasts may also participate in macrophage functional tuning. Fibroblasts provide a set of niche factors that rewire phenotypic balance of macrophages, also leading to their enhanced recruitment. Presence of fibroblasts shapes the transcriptional profile of macrophages in the presence of inflammatory cytokine signaling. Though this effect was described for RA-specific macrophage subset, a bidirectional interaction with fibroblasts may be expected, which can also contribute to functional polarization in OA. A recent study reported presence of synovium-specific signals produced by fibroblasts and highlighted the importance of modeling the relevant cellular microenvironment for macrophage phenotype studies [69]. In turn, synovial macrophages depending on phenotype may promote either invasive behavior of fibroblasts, as was clearly confirmed for HBEGF+ population in RA [51], or fibroblast repair responses by increasing the expression of TGF-β and collagen genes [54]. Studies in mice demonstrated that synovial fibroblasts are not a homogenous population, but contain morphologically and functionally distinct subsets with non-overlapping functions [70]. Macrophage–fibroblast interactions present an interesting area of research, that was recently reviewed elsewhere [69].

Targeting functional macrophage polarization may be an effective approach for pain control. Currently, most OA patients continue to present with pain even after available conventional drug treatment and, in some cases, also after total joint replacement [4], underscoring the urgent need to better understand the mechanisms of OA-related pain and develop new effective treatments for pain management. OA pain might be caused by a variety of mechanisms, including activation of nociceptive pathways by neuromodulator mediators, direct effects of cytokines and chemokines on neurons, or might occur as a result of infiltration of the spinal cord by immune cells [4]. Although radiographical and MRI studies have not shown correlation between pain and structural damage [71,72] or inflammation [73], it is now clear that the crosstalk between the immune system and nociceptors is fundamental to the development of chronic and acute inflammatory pain [74]. Immune pathways become activated early at pain onset and continue to develop during the later phases. In pain signaling, macrophage–neuron communications occur at different levels: in the affected joint through activation of nerve endings of nociceptors and synovial cells, in the dorsal root ganglia (DRG) where the cell bodies of nociceptors are clustered, and in the dorsal horn, where microglia can modify synapses between nociceptors and second-order neurons. Joints are densely innervated with sympathetic neurons, and many types of immune cells can play a role in neuroimmune crosstalk, including dendritic cells, neutrophils, macrophages, mast cells, and T cells. The role of bidirectional communication between nociceptors and macrophages in the context of OA is the most extensively studied [74]. Macrophages release an array of mediators, including IL-1β, TNFα, and neurotrophin, that bind to receptors on nociceptors, activating signaling pathways that lead to increased excitability and pain sensitization [4]. Neuroimmune interactions are not only important for pain development, but also for macrophage polarization and infiltration. In the context of joint damage and inflammation, nociceptors can directly respond to DAMPs, such as S100A8 and α2 -macroglobulin, to produce macrophage attracting chemokines [74,75]. Activated nociceptors produce a range of neuropeptides (substance P, CGRP, and others) that may modulate macrophage phenotypes. The activity of substance P binding to its receptor neurokinin-1 expressed on macrophages is a known example of M1-driving stimulus. Macrophages respond to substance P with NF-κB-driven production of IL-1β and TNF-α [74]. Nociceptors can also produce pro-M2 factors. Calcitonin gene-related peptide (CGRP) release promotes regulatory macrophage phenotype by stimulation of IL-10 production and inhibition of pro-inflammatory cytokine response. Communication between neurons and macrophages may also occur via exosome release. Exosomes may be an important source of micro-RNA that influence macrophage activity and phenotype [74]. For example, nociceptors secrete exosomes containing miR-21, a critical positive regulator of the NF-κB pathway and NLRP3 inflammasomes, and phagocytosis of miR-21-exosomes by macrophages drives M1 shift. However, this mechanism was observed in DRG neurons following peripheral axon injury [76]. 

Animal studies suggest macrophage targeting may be an effective approach for pain control. In a rat monoiodoacetate-induced (MIA) model of advanced knee OA characterized by resistance to COX inhibitors and dexamethasone, intravenous injection of clodronate-laden liposomes led to a two-fold decrease in the number of synovial macrophages and pain suppression [77]. Joints of CCL2^−/−^ and CCR2^−/−^ mice in DMM model showed decreased macrophage markers compared with wildtype mice and subsequently developed less pain despite a similar level of joint damage [78]. In another murine collagenase-induced OA model, the described GM-CSF/Jmjd3/interferon regulatory factor 4 (IRF4)/CCL17 axis of pain development included macrophages as the only cell population capable of CCL17 expression [79]. While CCL17 is known to be a chemokine with shared expression by M2 and M1 cells, GM-CSF is a classic M1 polarizing factor, which indicates that M1 polarization may participate in pain development. Mice deficient for *Irf4*, *Ccl17*, and *Ccr4*, but not for *Tnf* gene, showed reduced cartilage destruction, osteophyte size, and pain [79]. Treatment with anti-CCL17 monoclonal antibodies at the time of pain onset provided for rapid reversion of pain with sustained analgesic effect during the whole course of therapy. Treated mice had significantly milder disease and reduction in osteophyte size compared to untreated control. Targeting of *Jmjd3* was also efficient for pain control in this model [79]. Macrophages present in knee-innervating lumbar level DRG also display a proinflammatory phenotype, as demonstrated in a rat antigen-induced arthritis (AIA) model [74]. Macrophage infiltration of the DRG has been reported in experimental OA induced by DMM, starting around eight weeks after surgery. This coincided with increased gene expression of *Ccl2* and *Ccr2* mRNA in the DRG, and the onset of movement-provoked pain behavior [74]. Mice deficient for the *Ccr2* gene did not show sustained allodynia past week 8 after surgery, and did not develop movement-associated pain behavior, although presented with joint damage comparable with that in wild-type mice. At the same time, macrophages may have a positive role in pain resolution. Depletion of peripheral monocytes/macrophages from mice delayed resolution of intraplantar IL-1β- and carrageenan-induced inflammatory hyperalgesia. This effect depended on macrophage production of IL-10 and its signaling [80]. 

Transient receptor potential vanilloid 1 (TRPV1) is one the receptors involved both in pain sensing and macrophage polarization, relevant for human OA and rat OA models. TRPV1 is a nonspecific cation channel that may be activated by thermal, mechanical, and chemical stimuli. TRPV1 expression is increased two-fold in inflamed synovium of OA patients, where it colocalizes with M1 macrophage markers [81]. In a rat model, intra-articular injection of a TRPV1 agonist decreased synovitis scores along with a significant reduction of M1 cells (CD14^+^, CD80^+^, iNOS^+^, TRPV1^+^). In vitro TRPV1 activation had an anti-proliferative effect and reduced migration of M1 polarized cells. Suppression of M1 polarization was mediated by TRPV1-dependent Nrf2 activation [81]. Intra-articular injection of TRPV1 agonist CNTX-4975 (Centrexion Therapeutics) was studied in a Phase IIb trial in OA patients with chronic OA-associated knee pain and showed a reduction of pain scores. A Phase III trial for chronic moderate-to-severe OA knee pain was completed in June 2021 (results not published), and two other Phase III trials are currently ongoing (NCT03429049, NCT03661996). 

Mammalian target of rapamycin (mTOR) is a central regulator of cellular metabolism that has a key role in macrophage polarization. mTOR exists in two complexes, mTORC1 and mTORC2, with Tuberous sclerosis complex 1/2 (TSC1/2) being a negative upstream regulator of mTORC1. Mice with mTORC1 constitutive activation in the myeloid lineage due to TSC1/2 deletion are refractory to IL-4-induced M2 polarization, and develop hyperinflammatory response to M1 stimuli [32]. Activity of mTORC1 in macrophages was significantly enhanced in CIOA model, while being almost undetectable in normal synovium [32]. TSC1 knock-out mice demonstrated significantly higher OA and synovitis scores than controls, and had accelerated osteophyte formation. Analysis of the M1-associated transcriptional pattern revealed marked upregulation of Rspo2, a known Wnt signaling agonist, involved in chondrocytes and osteoblast differentiation. M1 macrophages secrete Rspo2 to induce terminal differentiation of articular chondrocytes, degradation of matrix proteoglycan and cartilage. Anti-Rspo2 antibody was injected intra-articularly to TSC1-deficient mice in CIOA model and showed efficacy in reduction of cartilage degeneration and OARSI score [32]. The opposite results were observed in mice with conditional ablation of the *Rheb1* gene, that resulted in inhibition of mTORC1 activity and upregulation of M2 response. Despite these promising results, the perspective of mTOR targeting in OA remains unclear because of contradictory results showing that inhibition of mTOR did not reverse the M1 phenotype in TSC1/2 knock-out cells, and that impaired mTOR pathway activation may favor M1 polarization [82,83], though these findings were made in a different context than OA.

Current OA treatments act at least in part through direct or indirect modulation of macrophages. Non-steroidal anti-inflammatory drugs (NSAIDs) exert their anti-inflammatory effects by inhibiting cyclooxygenase (COX) enzymes expressed by macrophages. COX-2 inhibitors were shown to decrease the level of pro-inflammatory M1 cytokines in IFP, with increased expression of M2 markers IL-10 and CD163, though this effect was present only in a subgroup of patients [40].

Corticosteroids are a class of steroid hormones with a powerful anti-inflammatory effect. Their use in OA is usually restricted to intra-articular injections for short periods, since prolonged treatment with corticosteroids can induce extreme immune suppression and bone resorption in the joint. Corticosteroids have cell-type-specific activities with multiple effects on T, B, dendritic, NK cells, and macrophages, as well as on chondrocytes, fibroblasts and other joint tissue cells. Among all these populations, synovial macrophages are considered to be the major targets of corticosteroids in arthritis [84]. Glucocorticoid therapy is associated with a substantial decrease in the number of macrophages in synovial tissues and induction of anti-inflammatory macrophage phenotype characterized by increased phagocytosis, decreased adhesiveness, and a severe attenuation in expression of classical pro-inflammatory cytokines, such as TNF, IL-1β and IL-6 [84].

Intra-articular administration of hyaluronic acid (HA), a natural glycosaminoglycan widely distributed throughout the body, has shown relative efficacy in some cases of OA. Macrophages undergo phenotypic changes depending on molecular weight of hyaluronan [85]. Low-molecular-weight HA possesses pro-inflammatory effects acting through TLR2 and TLR4, which is confirmed by up-regulation of pro-inflammatory genes, including NOS2, TNF-α, IL-12β, CD80, and enhanced secretion of NO and TNF-α. Therapeutically injected high-molecular-weight hyaluronic acid (HMW-HA) has demonstrated anti-inflammatory activity with increased expression of M2 markers, such as ARG1, IL-10, and MRC1 [85,86]. In murine DMM OA model, a hyaluronan derivative with extended joint residency (Hymovis^®^) did not result in significant change in the percentage of F4/80+ macrophages identified as M1 (CD11C^+^) or M2 (CD206^+^) subtypes, though the total amount of synovial monocyte/macrophage cells decreased [87]. There was a 25% increase in the percentage of M2c macrophages (F4/80^+^ CD206^+^ CD301^+^) considered to be an anti-fibrotic wound healing subtype within the M2 population [87]. However, the relevance of this population in human OA is unknown.

Stimulation of M2 polarization is a proposed mechanism of activity of TissueGene-C (tonogenchoncel-L), developed by Kolon TissueGene Inc.-a novel gene and cell therapy consisting of human allogeneic chondrocytes and irradiated GP2-293 (HEK-derived cell line) cells overexpressing TGF-β1. A Phase III trial of this therapy for OA is currently ongoing in the US (NCT03203330). Clinical data support TissueGene-C as a disease-modifying (DMOAD) candidate. In rat MIA, model expression of M2 macrophage markers Arg1, CD163, interleukin 10 receptor alpha subunit (IL-10RA), and heme oxygenase 1 (Hmox-1) were significantly upregulated upon treatment, and the level of IL-10 in SF achieved more than a four-fold increase [88]. There was a dramatic shift in the M1/M2 ratio in the synovium analyzed by the expression of CD86 and ARG1. Observed changes were accompanied by pain relief and structural improvement [88]. Several other studies described modulation of macrophage phenotypes via stem cell-based therapies. Both injection of cells as well as stem-cell-derived exosomes were shown to induce immunosuppressive macrophage phenotypes with decrease in M1 markers [89,90,91].

Recently, cell-tree therapeutic approaches targeting inflammatory signaling in OA have been developed, exploring the potential of small non-coding RNAs [92]. The involvement of small non-coding RNAs in OA is currently well-known. Micro RNAs (miRNA) can suppress the expression of certain proteins, while circular RNA (circRNA) can interact with them in a sponging manner, therefore inhibiting their activity. This regulatory non-coding RNA signaling network can regulate cartilage destruction through the expression of matrix degrading enzymes. For instance, it was shown that inhibition of miR-127-5p by circRNA.33186 aggravated OA in a DMM model [93]. Another study has shown the protective role of circSERPINE2 RNA through inhibition of miR-1271 [94]. Although less is known on the role of non-coding RNA in inflammation signaling, several promising RNA species have been identified. It was found that miR-214-3p is down-regulated in OA joints and replenishment of this protective miRNA helped alleviate symptoms in a murine model [95]. Circular RNA has_circ_0005567 was found to act at the level of macrophage polarization, promoting M2-like differentiation of synovial macrophages through inhibiting miR492 and suppressing OA progression [96]. In light of these animal studies, intraarterial injection of small non-coding RNA targeting a specific mechanism of cartilage destruction and inflammation appears to be a promising approach for therapy development. However, more studies are needed to translate these findings into clinical practice. 

Macrophage reprogramming may also be achieved by metabolic modulation, as was shown in studies on ZIF-8 nanoparticles (NPs) loaded with iNOS inhibitor and catalase and additionally modified by anti-CD16/32 antibody to prolong the retention time in joints [97]. Based on qRT-PCR in vitro results, modified ZIF-8 NPs reduced M1-related genes, including IL1β, IL12β, IL6, TNFα, and iNOS/Arg-1, and decreased the percentage of CD206-negative and CD16/32-positive cells (M1 macrophages) from 27% to 14%. In vivo, ZIF-8 NPs were studied in mice with ACLT, a model characterized by a higher inflammatory component than DMM model. ZIF-8 NPs were able to reduce the synovitis, as demonstrated by reduced angiogenesis and M1 infiltration. Additionally, NPs up-regulated synovial M2 phenotype macrophages (CD206-positive cells), led to decreased chondrocyte apoptosis and joint structural improvement assessed by X-ray [4].

## 5. Conclusions

Currently, clinical success in OA treatment remains marginal, with no treatment able to stop or reverse OA progression. One of the unsolved problems in the development of effective OA treatment is the need for identification of selective disease time-points and personification of therapeutic strategy corresponding to the pathological stage and heterogeneity of disease. A total of six major OA clinical phenotypes have been proposed, though they were insufficient to classify all the OA patients [98]. Irrespective of the multifaceted nature of OA pathophysiology, it is now accepted that inflammation plays an important role in OA onset and progression at least in a subset of patients and could be a potential target for the development of disease-modifying osteoarthritis drugs (DMOADs).

The progress in the development of macrophage-targeting strategies in OA is complicated due to a number of currently unanswered questions. First, macrophage transcriptome studies highlighted the limitations of the M1/M2 paradigm and the need for more detailed evaluation of macrophage functional phenotypes. At present, clear classification of functional macrophage subpopulations in OA is lacking. The scRNA-seq approaches can help explore macrophage heterogeneity, but warrant additional studies to understand how these transcriptionally defined clusters relate to macrophage functional phenotypes and clinical symptoms. Second, the understanding of macrophage heterogeneity in OA and development of therapeutic strategies corresponding to exact immune profile critically depend on identification and validation of biomarkers, specific for distinct macrophage subgroups. The classification of macrophages is not an easy task, as the majority of markers are not exclusive for one phenotype and different signatures do not necessarily exclude each other and often coexist. Third, macrophage phenotypes are plastic and rapidly adopt to local signals. Effective therapeutic modulation of macrophage plasticity requires understanding of the complex microenvironment in the inflamed joint, as well as its changes during the course of OA progression. Finally, inflammatory players and processes differ greatly between patients, and the role of macrophages in OA pathogenesis may vary depending on the stage and endotype of the disease. The development of an OA classification system based on immunopathological patterns is a critical necessity for further studies.

## Figures and Tables

**Table 1:** Summary of macrophage populations identified in human knee OA.

Macrophage Subpopulation	Surface Markers/Gene Expression	Clinical Correlates, Proposed Function	Reference
Synovial macrophages	HLA-DR^+^, CD14^+^, CD64^+^, CD11c^+^, CD86^+^, FOLR2^+^	iOA and cOA subgroups did not correlate with radiographic measures of disease severity (Kellgren–Lawrence X-ray score), indicators of systemic inflammation, medication	[48]
Synovial macrophages with pro-inflammatory phenotype (iOA)	Ki67^high^, E2F8^high^, CDT1LK1^high^, CTRL^high^		
Synovial macrophages with classical phenotype (cOA)	Ki67^low^, IGFBP5^high^, HTRA1^high^, EFEMP1^high^, SMAD3^high^, RUNX2^low^		
Resident macrophages from meniscal tissue	SIGLEC1^low^, CSF2^low^, TNF^low^	ND	[50]
Synovial macrophages specific cluster	CD14^+^ ITGB5^+^, ADORA3^+^, MERTK^+^	Functional polarization distinct from both M1/M2	[51]
M1	CD11c^+^ C86^+^	Increase in M1/M2 ratio in human knee OA was shown to positively correlate with Kellgren–Lawrence grade	[39]
M2	CD206^+^ CD163^+^		
Synovial macrophages with inflammatory phenotype (I-MΦ)	HLA-DRA^high^, CD14^high^, CD16^high^, IL1A^high^, IL1B^high^, IL6^high^, TNF^high^, CCL2^high^ CCL3^high^	Proposed to be the main source of inflammatory cytokines	[29]
Synovial macrophages with immunoregulatory phenotype (IR-MΦ)	HLA-DRA^high^, CD14^high^, CD163^high^, STAB1^high^, TNXIP^high^, CD169^high^	Proposed immunosuppressive function	[29]
Resident synovial macrophages	MHCII^−^, TREM2^+^, tight junctional proteins	Proposed barrier function	[52]
Synovial macrophages SC-M1	CD11c^+^ CD38^+^ IL1B^+^, NR4A2^+^, HBEGF^+^, PLAUR^+^ IFITM3^+^	Expression profile matches mouse monocyte-derived synovial macrophages, proposed pro-inflammatory activity	[53]
Synovial macro-phages SC-M2	CD14^+^ CD11c^+/−^CD38^−^ VSIG4^+^, GPNMB^+^, MERTK^+^, NUPR1^+^, CTSK^+^, HTRA1^+^	Expression profile matches resident synovial macrophages identified in mice	[52,53]
Synovial macrophages SC-M3	CD11c^+^ CD38^+^ C1QA^+^, MARCO^+^	Expression profile matches resident synovial macrophages identified in mice	[52,53]
Synovial macrophages SC-M4	CD11c^+^ CD38^+^ SPP1^+^, type I and II IFN induced genes, including IFITM3 and IFI6	Expression profile matches mouse monocyte-derived synovial macrophages	[52,53]

## Data Availability

Not applicable.
